# A Cross-Taxon Analysis of Insect-Associated Bacterial Diversity

**DOI:** 10.1371/journal.pone.0061218

**Published:** 2013-04-16

**Authors:** Ryan Thomas Jones, Leticia Gonzales Sanchez, Noah Fierer

**Affiliations:** 1 Cooperative Institute for Research in Environmental Sciences, University of Colorado – Boulder, Boulder, Colorado, United States of America; 2 Miramontes Arts and Sciences Program, University of Colorado – Boulder, Boulder, Colorado, United States of America; 3 Department of Ecology and Evolutionary Biology, University of Colorado – Boulder, Boulder, Colorado, United States of America; Argonne National Laboratory, United States of America

## Abstract

Although it is well known that plants and animals harbor microbial symbionts that can influence host traits, the factors regulating the structure of these microbial communities often remain largely undetermined. This is particularly true for insect-associated microbial communities, as few cross-taxon comparisons have been conducted to date. To address this knowledge gap and determine how host phylogeny and ecology affect insect-associated microbial communities, we collected 137 insect specimens representing 39 species, 28 families, and 8 orders, and characterized the bacterial communities associated with each specimen via 16S rRNA gene sequencing. Bacterial taxa within the phylum Proteobacteria were dominant in nearly all insects sampled. On average, the insect-associated bacterial communities were not very diverse, with individuals typically harboring fewer than 8 bacterial phylotypes. Bacterial communities also tended to be dominated by a single phylotype; on average, the most abundant phylotype represented 54.7% of community membership. Bacterial communities were significantly more similar among closely related insects than among less-related insects, a pattern driven by within-species community similarity but detected at every level of insect taxonomy tested. Diet was a poor predictor of bacterial community composition. Individual insect species harbored remarkably unique communities: the distribution of 69.0% of bacterial phylotypes was limited to unique insect species, whereas only 5.7% of phylotypes were detected in more than five insect species. Together these results suggest that host characteristics strongly regulate the colonization and assembly of bacterial communities across insect lineages, patterns that are driven either by co-evolution between insects and their symbionts or by closely related insects sharing conserved traits that directly select for similar bacterial communities.

## Introduction

Insects play vital roles in the functioning of both natural and managed systems that include pollinating crops, spreading disease, altering soil fertility, and plant herbivory. The specific traits and ecological attributes are, in part, related to the microorganisms found within individual insects. Symbioses between particular insect species (e.g. aphids, tsetse flies, psyllids, termites) and their primary symbionts have been well documented [1], [2], but factors affecting the distribution of other insect-associated bacteria across insect diversity is not well understood.

Insect-associated bacterial communities are a mix of mutualist, pathogenic, and commensal bacteria. Insect diets are often nutrient-poor or incomplete diet, and associated bacteria can aid their survival by synthesizing essential nutrients. Some insects have specialized cells called bacteriocytes that house beneficial intracellular bacteria thought to enhance nutrient-poor diets [3]. The obligate symbiosis between aphids and *Buchnera aphidicola*, for example, has been explored intensively, and genome studies revealed that *B. aphidicola* provide essential metabolic functions for the aphid host [4], [5], [6]. In addition to providing nutrients, bacteria can increase insect fitness through a suite of other mechanisms, such as protection against pathogenic viruses [7], [8], protection against trypanosomatid parasites [9], tolerance to heat stress [10], resistance to parasitoid wasps [11], resistance to pathogenic fungi [12], induced color changes for camouflage [13], and the production of sex and aggregation pheromones [14], [15], [16]. Other bacteria are insect pathogens: spore-forming bacilli are consumed by insects and produce lethal toxins [17]; *Pseudomonas* spp., *Streptococcus* spp. and *Enterobacter* spp. are common pathogens that presumably gain access to insects via their digestive tract [18], [19]; and insect predators (e.g. nematodes) can harbor symbionts that become insect pathogens after the predator attacks and infects its prey [20].

The ecological factors regulating bacterial assemblage patterns within individual insect species have recently been explored by harnessing the power of high-throughput DNA sequencing techniques. Bacterial communities of fleas were found to differ across time and space [21], aphid-associated bacteria differed between two co-occurring species and among sub-populations within species [22], and mosquito-associated bacteria differed across life stages and diet types [23]. These studies have elucidated how bacterial communities can shift in composition across populations of a given insect species. However, there have been few cross-taxon studies, making it difficult to identify the general factors that govern bacterial community assemblage patterns across a wide range of insect species.

Recent cross-taxon comparisons of insect-associated microbial communities have aimed to disentangle host and diet effects on microbial composition. In ants, microbial communities differed among herbivorous and predatory ants, but were similar among species within the same trophic level [24]. In laboratory experiments, *Drosophila*-associated microbial communities changed in response to diet changes and microbial communities did not differ among species when fed the same diet [25]. These studies suggest that diet can affect microbial communities, at least among closely related species. To our knowledge, only a single meta-analysis has attempted to compare the structure and composition of bacterial communities across a broad diversity of insect species [26]. This study of 62 insect species from seven orders found that insect diet and taxonomy influence the microbial community, though the effects of each were marginal. Although this was an impressive study, it was limited in its ability to quantify cross-taxon differences in microbial communities due to high variability in the number of sequences per sample and differences in the molecular techniques used to generate data among the various original studies included in the analysis. Furthermore, since bacterial communities from the individual insects were combined, Colman *et al*. were not able to quantify how the intra-specific variability compares to the inter-specific variability in microbial community composition across insect taxa.

Here we used a cross-taxon approach to determine the effects of host and diet on insect-associated bacterial communities, but have designed the study to expand upon previously-published work by collecting all samples from one location at one time, surveying the microbial community in each collected sample to an equivalent depth using the same method, and analyzing multiple specimens per insect species. We conducted our survey to characterize bacterial communities of a large number of insect species, determine richness and evenness of insect-associated bacterial communities, investigate the effects of diet and insect taxonomy on bacterial community composition, and explore cross-taxon community assemblage patterns.

## Materials and Methods

### Insect Collections and Identifications

To minimize the effects of climate, biogeography and temporal shifts within an insect population, insects were intensely sampled over a short period from a single location. Insects were collected using a variety of techniques (light traps, sweep netting, beat sheeting, active capture, and pit-fall traps) from March 3– March 7, 2010 in Honolulu, Hawaii. The vast majority of specimens were collected at the Lyon Arboretum, but a few specimens were also collected from the University of Hawaii at Manoa and the Hawaii Humane Society. Upon capture, insects were immediately placed in 70% ethanol, and then stored at −20°C from the end of the collection day until DNA extraction. Only adult insects were included in the study. Most specimens were classified to species level (74.4%) and all specimens were classified to the genus level based on morphology (Table 1). A voucher specimen exists for every species with at least two collected representative samples – if only one specimen was collected, it was used for molecular analyses and no voucher specimen exists.

**Table 1:** List of insect specimens.

Order	Family	Genus species	Diet	Number	Code
Coleoptera	Anthribidae	*Aerecerus levipennis*	D	5	CAAl
Coleoptera	Coccinellidae	*Cryptolaemus sp.*	P	6	CCCs
Coleoptera	Coccinellidae	*Hippodamia sp.*	P	1	CCHs
Coleoptera	Coccinellidae	*Nephus sp.*	P	2	CCNs
Coleoptera	Cucujidae	*Cryptomorpha desjardinsi*	FR	4	CUCd
Coleoptera	Scarabidae	*Adoratus sp.*	FR	1	CSAs
Diptera	Ceratopogonidae	*Atrichopogon jacobsoni*	Ha	5	DCAj
Diptera	Ceratopogonidae	*Forcipomyia hardyi*	Ha	2	DCFh
Diptera	Chloropidae	*Rhodesiella scutellata*	NP	4	DHRs
Diptera	Culicidae	*Aedes albopictus*	HN	5	DUAa
Diptera	Culicidae	*Culex quinquefasciatus*	HN	2	DUCq
Diptera	Drosophilidae	*Drosophila immigrans*	D	10	DDDi
Diptera	Drosophilidae	*Drosophila sulfirigaster*	D	5	DDDs
Diptera	Lauxaniidae	*Homoneura unguiculata*	D	5	DLHu
Diptera	Muscidae	*Atherigona hendersoni*	O	1	DMAh
Diptera	Neriidae	*Telostylinus lineolatus*	D	5	DNTl
Diptera	Psychodidae	*Pyschoda sp.*	U	2	DPPs
Diptera	Sciaridae	*Bradysia sp.*	D	3	DSBs
Diptera	Syrphidae	*Allograpta sp.*	NP	1	DYAs
Hemiptera	Anthocoridae	*Orius persequens*	P	5	HAOp
Hemiptera	Aphididae	*Aphis gossyppii*	LX	3	HPAg
Hemiptera	Aphididae	*Pentalonia caladii*	LX	5	HPPc
Hemiptera	Cercopidae	*Philaenus spumarius*	LX	5	HCPs
Hemiptera	Cicadellidae	*Sophonia rufofascia*	LX	4	HASr
Hemiptera	Cimicidae	*Cimex lectularius*	Ha	5	HMCl
Hemiptera	Lygaeidae	*Nysius communis*	LX	1	HLNc
Hemiptera	Lygaeidae	*Nysius palor*	LX	2	HLNp
Hymenoptera	Apinae	*Apis sp.*	NP	5	YAAs
Hymenoptera	Colletidae	*Hyleus sp.*	NP	1	YCHs
Hymenoptera	Formicidae	*Cardiocondyla emeryi*	O	4	YFCe
Hymenoptera	Formicidae	*Leptogenys falcigera*	O	2	YFLf
Hymenoptera	Formicidae	*Paratrechina bourbonica*	O	5	YFPb
Hymenoptera	Formicidae	*Pheidole megacephala*	O	1	YFHm
Hymenoptera	Xylocopinae	*Xylocopa sp.*	NP	4	YXXs
Isoptera	Termitidae	*Coptotermes formosanus*	DX	6	ITCf
Lepidoptera	Geometridae	*Macaria abydata*	FR	1	LGMa
Neuroptera	Chrysopidae	*Chrysopa microphya*	O	2	NCCm
Siphonoptera	Pulicidae	*Ctenocephalides felis*	Ha	5	SPCf
Siphonoptera	Pulicidae	*Echidonphaga gallinacea*	Ha	2	SPEg

Diet: D, Detritivorous; DX, Dead-wood xylophagus; FR, Foliage and Roots; Ha, Haematophagous; HN, Haematophagous/Nectarivorous; LX, Live-plant xylophagus; NP, Nectarivorous/Pollenivorous; O, Omnivorous; P, Predacious.

### Bacterial Community Analyses

Prior to DNA extraction, we rinsed insect samples with 100% ethanol to minimize the contribution of bacteria from insect surfaces. We extracted DNA from insect samples using the MoBIO PowerSoil-htp 96 Well DNA Isolation kit (Carlsbad, CA). For most samples, DNA was extracted from whole insects, but very large insects were frozen in liquid N2 and pulverized with a mortar and pestle to obtain a homogenous tissue sample of an appropriate size. We did not attempt to distinguish between those bacteria that are endosymbionts and those that are found within insect guts. We used barcoded pyrosequencing of a portion of the 16S rRNA gene to characterize bacterial communities. We amplified each sample (n = 137) in triplicate using 5 PRIME MasterMix with bacterial 16S rRNA gene primers that amplify the V1 and V2 hypervariable regions. The forward primer (5′-GCCTTGCCAGCCCGCTCAGTCAGAGTTTGATCCTGGCTCAG-3′) contains the 454 Life Sciences primer B sequence, the 16S rRNA gene 27 f primer, and a two-base ‘TC’ linker; the reverse primer (5′-GCCTCCCTCGCGCCATCAGNNNNNNNNNNNNCATGCTGCCTCCCGTAGGAGT-3′) contains the Life Sciences primer A sequence, a 12-bp error-correcting barcode, the 16S rRNA gene 338 r primer, and a two-base ‘CA’ linker [27]. Amplifications occurred under the following conditions: 94°C for 5 min; 40 cycles of 94°C for 45 s, 50°C for 30 s, 72°C for 90 s; 72°C for 10 min. PCR products from each of the three independent reactions were combined and then cleaned using the MoBIO UltraClean-htp 96 Well PCR Clean-Up Kit (Carlsbad, CA). We estimated the DNA concentrations of clean PCR products from individual samples using the Invitrogen Quant-IT PicoGreen dsDNA Assay kit (Carlsbad, CA). DNA sequencing was conducted at EnGenCore (Columbia, South Carolina) on a Roche Genome Sequencer running the GS FLX Titanium chemistry.

### Data Processing and Analysis

We processed DNA sequence data using QIIME v1.5 [28]. On average, 2,525 DNA sequences were obtained per specimen (minimum = 61; maximum = 4,115; standard deviation = 685). Sequences were truncated to 275 basepairs in length and low-quality sequences were removed using QIIME’s default settings. Sequences were binned into phylotypes (a species surrogate for microbial lineages) based on a 97% sequence similarity criteria using the uclust setting, and the most abundant sequence in each defined phylotype was selected as its representative sequence. To correct for over-inflation of diversity estimates due to sequencing error [29], [30], we removed phylotypes from individual samples that did not represent at least 1% of community membership within that sample. Any sample with less than 500 quality DNA sequences was removed from the dataset. Subsequently, all samples were rarefied to a set sequencing depth of 500 randomly selected reads per sample prior to all downstream analyses. Our final dataset included 477 unique bacterial phylotypes and these data have been deposited in GenBank (Accession numbers: HE660361– HE661175).

The number of unique phylotypes detected in each sample was used as an estimate of richness in individual samples, and Shannon’s Evenness and Simpson’s Diversity were used to assess bacterial community evenness for each insect specimen. Representative sequences for each phylotype were classified according to the RDP classifier (http://rdp.cme.msu.edu) and aligned using the NAST aligner [31] on the Greengenes webserver (http://greengenes.lbl.gov). Aligned sequences were used as input for the generation of a neighbor-joining tree in FastTree [32].

Similarity of bacterial communities between samples (beta diversity) was quantified using a metric based on phylotype abundances (Bray-Curtis Dissimilarity) and using phylogenetic metrics based upon the amount of unique phylogenetic diversity found in each sample (unweighted and weighted UniFrac) [33], [34]. We used an Analysis of Similarity (ANOSIM) procedure in the QIIME analysis package to test for significant effects of insect taxonomy (species, family, and order). We also used an ANOVA to assess the effects of taxonomy (categories: within insect species, within insect families/among species, within insect orders/among families, and among orders) and diet (categories: within species, among species/within diet classification, and among diet classifications) on bacterial community dissimilarity. Insects were classified into nine diet types: detritivorous, dead-wood xylophagous, foliage/roots, haematophagous, haematophagous/nectarivorous, live-plant xylophagous, nectarivorous/pollenivorous, omnivorous, or predacious. To visualize clustering patterns of bacterial communities among insect species, we performed a Bray-Curtis transformation of the average phylotype abundances for each insect species and used the UPGMA clustering procedure in QIIME.

## Results

We characterized the bacterial communities of 137 individual insect samples representing 39 insect species, 28 insect families, and 8 insect orders (Table 1). In total, we identified 477 unique bacterial phylotypes, with phylotypes defined at the 97% sequence similarity level.

**Figure 1:**
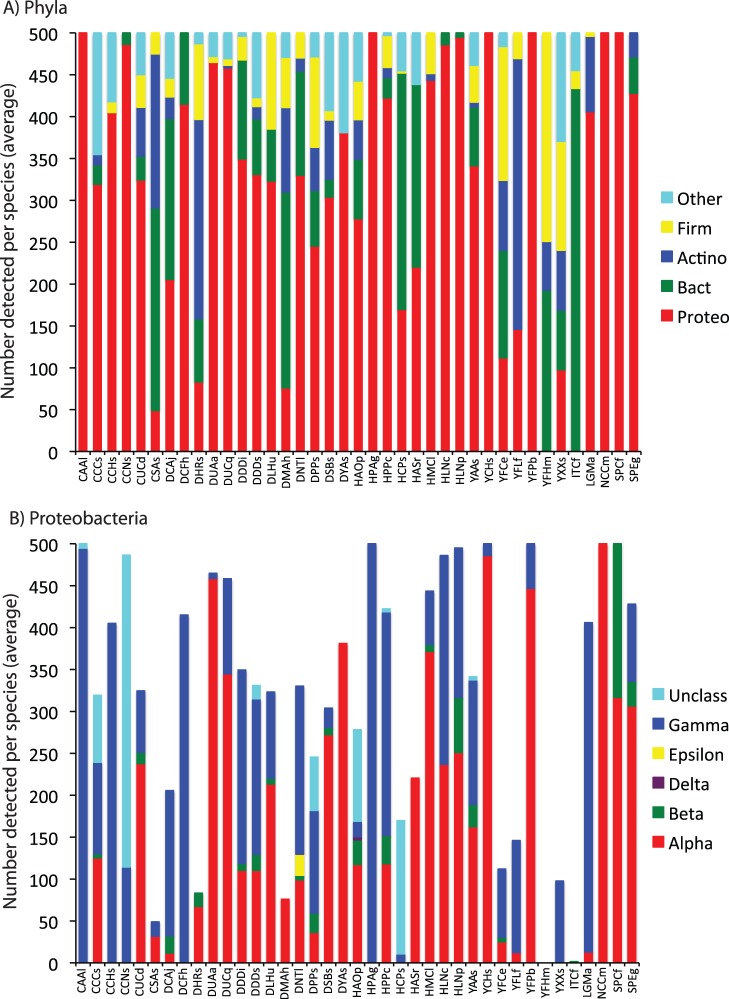
Classification of bacterial community members for each insect species. Values are averaged across all samples within the species. Bacteria are classified to phylum (Firm = Firmicutes; Actino = Actinobacteria; Bact = Bacteroidetes; Proteo = Proteobacteria) (A). Proteobacteria are classified to sub-phylum (B). Each column is an insect species, which are subsequently grouped according to insect family and insect order. Four letter codes for insect species are detailed in Table 1.

Taxonomic classification of these phylotypes revealed that bacterial communities were dominated by only a few phyla, with greater than 94% of community membership represented by four phyla (average relative abundance values averaged across all insect species): Proteobacteria (64.6%), Bacteroidetes (14.9%), Actinobacteria (7.7%), and Firmicutes (6.9%) (Figure 1A). Proteobacteria were particularly abundant, with three sub-phyla dominating the insect-associated communities (again, average abundance values averaged across all species): Alphaproteobacteria (31.6%), Gammaproteobacteria (26.1%), and Betaproteobacteria (2.6%) (Figure 1B).

The bacterial diversity levels observed within individual insects (alpha diversity) were relatively low (Table 2). On average, individual insects harbored 7.5 unique bacterial phylotypes, with a range of 1 phylotype per insect to 20 phylotypes per insect (500 sequences analyzed per individual insect specimen). Individual insects tended to be dominated by a single phylotype with the most abundant phylotype representing 54.7% of bacteria, on average.

**Table 2:** Richness and evenness of bacterial communities for eight insect orders.

		Richness		Shannon’s Evenness		Simpson’s D	
	N	Avg. #	Error	Avg. E	Error	Avg. D	Error
Coleoptera	19	6.53	0.75	0.60	0.05	0.49	0.06
Diptera	50	8.50	0.58	0.71	0.04	0.35	0.04
Hemiptera	30	7.47	0.84	0.67	0.04	0.41	0.04
Hymenoptera	22	8.36	1.04	0.60	0.08	0.42	0.08
Isoptera	6	5.83	0.79	0.35	0.05	0.74	0.06
Lepidoptera	1	7.00	–	0.67	–	0.36	–
Neuroptera	2	1.50	0.5	0.05	0.05	0.99	0.01
Siphonoptera	7	5.71	0.75	0.48	0.08	0.61	0.09

**Figure 2:**
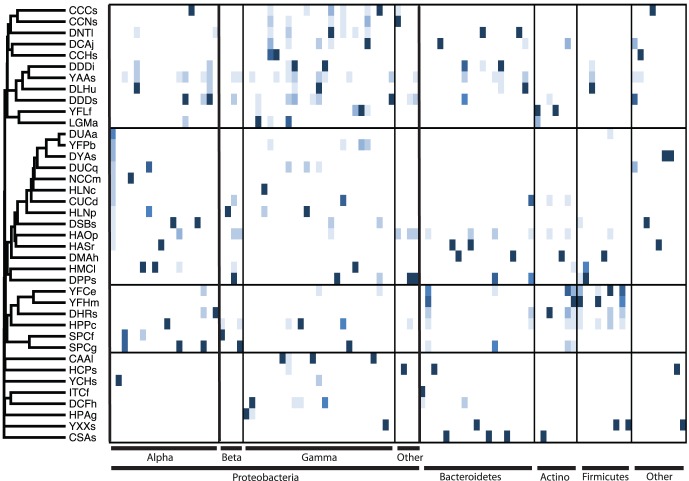
Bray-Curtis cluster of insect species based on their associated bacterial communities (all 477 bacterial phylotypes used for Bray-Curtis analysis) and Z-scores of the 96 most abundant bacterial phylotypes with lowest scores in light blue and highest scores in dark blue. Each column is a unique bacterial phylotype. Phylotypes are arranged according to taxonomic classification. Insect species are identified by a four-letter code (Table 1) with the first letter indicating the order, as follows: (C) Coleoptera, (D) Diptera, (H) Hemiptera, (I) Isoptera, (L) Lepidoptera, (N) Neuroptera, (S) Siphonoptera, and (Y) Hymenoptera.

Few bacterial phylotypes were shared across insect species and nearly every insect species harbored a very distinct bacterial community (Figure 2). Not only are the bacterial communities of individual insects typically dominated by only a few phylotypes (low alpha-diversity), but the distribution of phylotypes are generally limited to only a few insect species (high beta-diversity) (Figure 2). Of all 477 bacterial phylotypes, 69.0% were not shared among insect species (i.e. 69% were only detected in a single insect species). Few phylotypes were widely distributed among insect families, and only 5.7% (27/477) of bacterial phylotypes were detected in five or more insect species. The most cosmopolitan phylotype was a single *Wolbachia* phylotype that was detected in 43.6% (17/39) of insect species (Supplementary Table 1).

**Figure 3:**
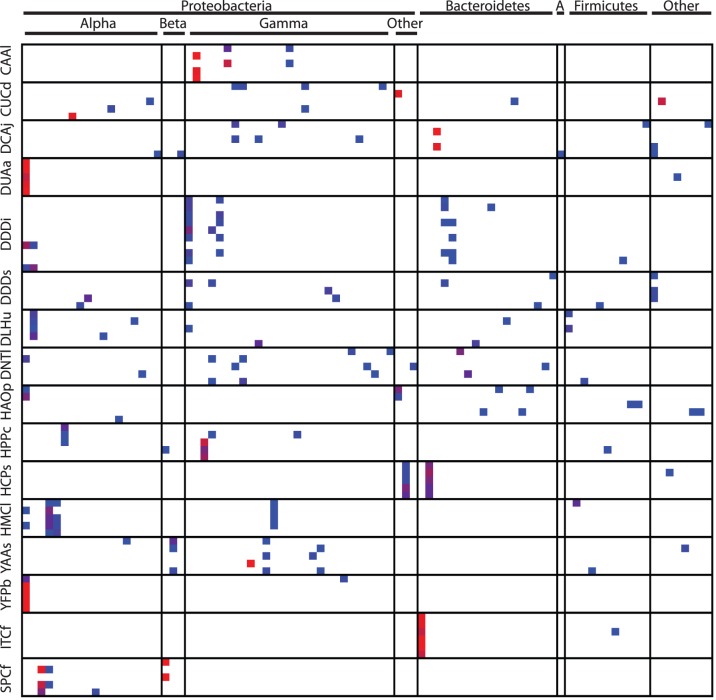
Frequencies of bacterial phylotypes in insect species with at least 5 specimens. Each row is an individual insect specimen and each column is a single bacterial phylotype. Insect codes are detailed in Table 1. Phylotypes with relative abundance of 10% or greater in any one insect are included. Absence of a phylotype within a specimen is indicated by white; relative abundance of a phylotype within a specimen ranges from blue (>0%–10%) to red (90%–100%).

**Figure 4:**
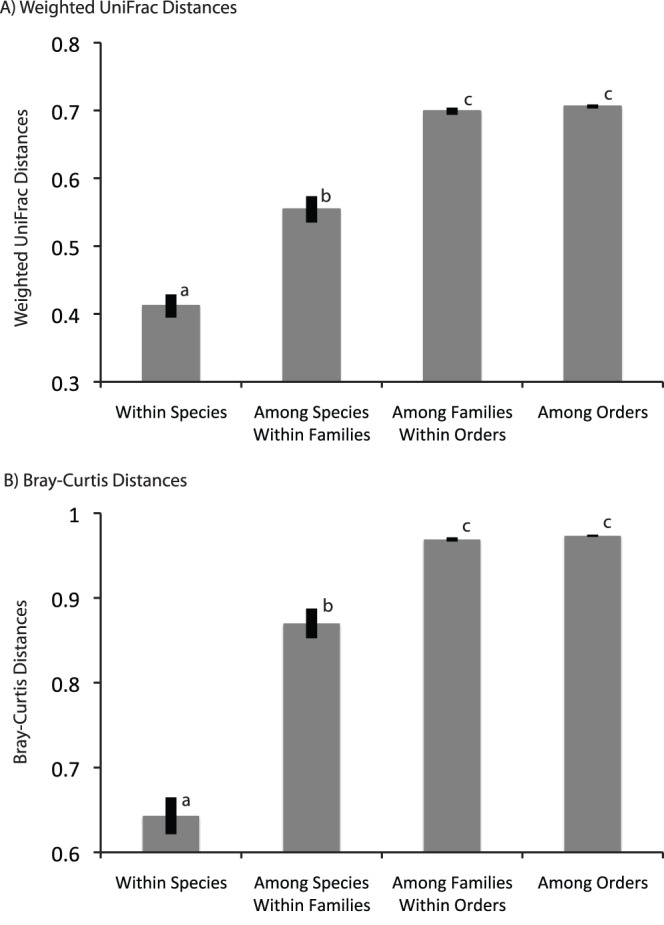
Effect of taxonomic classification on bacterial community dissimilarity. Average weighted UniFrac (A) and Bray-Curtis (B) dissimilarity values of bacterial communities from specimens within species, within families and among species, within orders and among families, and among orders. Error bars represent standard error.

**Figure 5:**
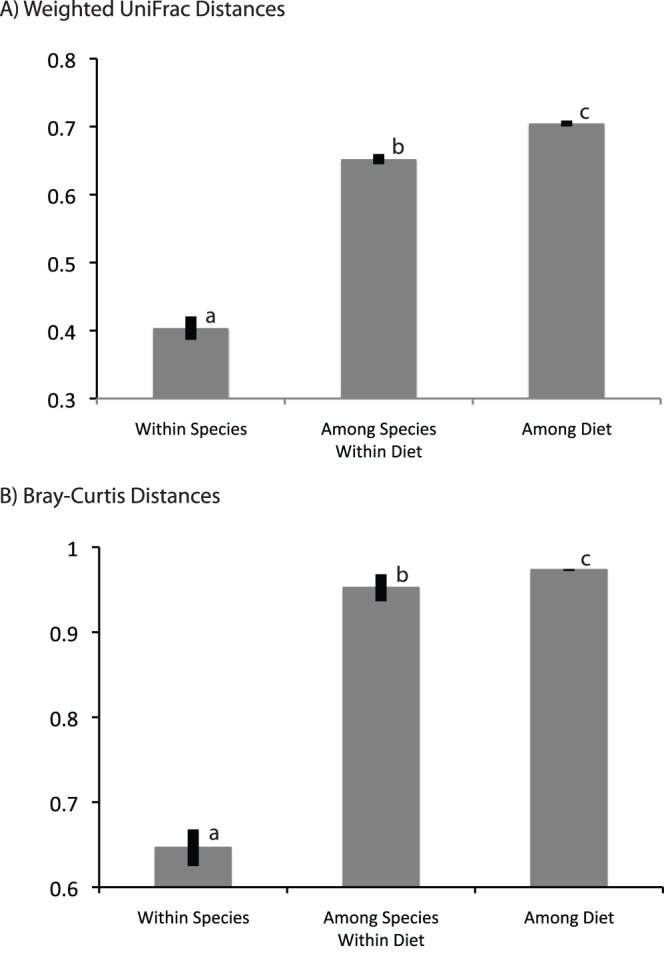
Effect of diet on bacterial community dissimilarity. Average weighted UniFrac (A) and Bray-Curtis (B) dissimilarity values of bacterial communities from specimens within species, among species/within diet classification, and among diet classifications. Error bars represent standard error.

In some insect species, the dominant bacteria were identical among individual specimens; in others, however, dominant phylotypes were not shared among individual specimens of the same species (Figure 3). Nevertheless, insect-associated bacterial communities were significantly more similar among closely related insects than among distantly related insects (Table 3). This relationship held across all taxonomic groupings tested (within insect species, families, and orders), but community similarity was less apparent at deeper taxonomic ranks (e.g. insect order) than at the finer-scale groupings (e.g. insect species) (Table 3, Figure 4). The analysis of similarity (Table 3) shows a greater effect of deeper taxonomic ranks on community similarity than the ANOVA (Figure 4) because fine-scale groupings are nested within the deeper ranks (e.g. within family comparisons compare all specimens within the family including those of the same species), whereas they are separated in the ANOVA. Diet also had a significant effect on bacterial communities composition (Figure 5), but this effect of diet was much lower in magnitude than the effect of insect taxonomy.

**Table 3:** Analysis of Similarity of bacterial communities across insect taxonomy.

	Unweighted		Weighted		Bray-Curtis	
	R	p-value	R	p-value	R	p-value
Species	0.717	<0.001	0.589	<0.001	0.699	<0.001
Family	0.482	<0.001	0.489	<0.001	0.528	<0.001
Order	0.126	<0.001	0.114	0.004	0.150	<0.001

## Discussion

The same four phyla that dominated the insect-associated communities (Figure 1A) are also those that constitute the majority of bacterial communities associated with humans [35], mammalian guts [36], [37], reptile guts (though Actinobacteria are rare) [38], amphibian skin [39], and coral (though Bacteroidetes are often rare) [40], [41], [42], [43]. The dominance of these four phyla across a wide range of animal-associated bacterial communities suggests that these phyla, or individual lineages within these phyla, have ecological attributes that allow them to frequently reside within animal hosts and may represent a broadly defined ‘core microbiome’ associated with animals. Proteobacteria account for most of the known primary insect symbionts and this likely explains the much higher abundance of Proteobacteria within insects relative to mammals. The high abundance of Proteobacteria in insects (Figure 1B) may be due to insects actively recruiting Proteobacteria or due to proteobacterial taxa being more effective than other bacterial groups at invading and proliferating within new insect hosts. These bacterial phyla are also common members of soil and marine bacterial communities [44], [45], [46], and their occurrence in soil, marine, and animal-associated bacterial communities is a testament to their phylogenetic and functional diversity.

Despite deep sequencing coverage (500 DNA sequences per sample), insect-associated bacterial richness was low (Table 2), and this result is consistent with other recent studies finding low bacterial richness in insects [22], [47], [48], [49]. Richness is far lower and less even than what is commonly observed in humans and other mammals. Lower bacterial diversity in insects relative to mammals has been noted previously [50], and may be explained by differences in the immune systems of vertebrates and invertebrates; the adaptive immune system of vertebrates may be more permissive of a diverse suite of microbes whereas the innate immune system of invertebrates may simply target the vast majority of microbial diversity [51]. Low diversity may also be a result of insects having specific structures (e.g. bacteriocytes) that are used to house large numbers of specific bacterial lineages. In either of these cases, low bacterial richness would be due to insect hosts directly regulating the colonization and assembly of bacterial communities.

An alternative explanation for low bacterial richness is negative interactions between established insect symbionts and invading bacteria. Commensal insect gut bacteria prevent pathogens and other bacteria from establishing [52]. Insect symbionts have frequently been found to limit the invasion of pathogenic viruses and other parasites [8], [9], [12], [53], [54], and these types of interactions also likely extend to environmental or pathogenic bacteria. In fleas, for example, the presence of *Bartonella* or a *Rickettsiales*-lineage significantly reduced overall bacterial community diversity [55]. Insects also harbor fungal symbionts [56], [57], [58], [59], and antagonistic interactions between associated fungi and bacteria may also limit bacterial diversity.

In some insect species, bacterial communities were remarkably similar among individual specimens; in other species, the dominant phylotypes differed among individuals (Figure 3). These contrasting patterns have also been observed in other studies of insect-associated microbial communities. For example, bees, *Apis millifera*, have a core set of bacterial taxa that are present in most individuals [48]. On the other hand, the bacterial communities associated with mosquitoes and fleas can vary substantially among individuals [49], [55]. However, even in insects that tend to have varied communities among individuals, the phylotypes causing the intra-specific variability tend to be species specific (Figure 3).

Few bacterial phylotypes (<6%) were detected in five or more insect species, highlighting the ability of a minority of bacterial taxa to overcome the barriers to dispersal and colonization affecting the majority of insect-associated bacteria. In general, individual insect species harbor a unique consortium of bacteria (Figure 2) and the high similarity of communities within insect taxonomic groups (Table 3, Figure 4) suggests either co-evolution between insects and their bacteria or that closely related insects share conserved traits that directly select for or permit invasion of similar bacteria. Numerous genomic studies have revealed obligate mutualisms between insects and their primary symbionts [60], [61], [62], [63], and these types of symbioses are likely widespread across insects. The similarities of bacterial communities among closely related insects is likely due to some combination of vertical-transmission of symbionts from mother to offspring, a higher likelihood of horizontal transmission of facultative symbionts among individuals within an insect species than among distantly related ones, and host-mediated selection.

Our finding that insect hosts affect bacterial community assembly (Table 3, Figure 4) is supported by recent cross-taxon investigations of other insect-associated bacteria. In fleas and ticks, the arthropod host governed bacterial assemblages whereas rodent host and environmental conditions did not [64]. A meta-analysis of 62 insect species also found insect taxonomy to significantly structure bacterial communities [26]. Colman *et al*. suggested that within-order variation among samples indicated that insect taxa did not maintain distinct bacterial communities. However, this interpretation may be biased by their focus on order-level taxonomy and lack of intra-family or intra-specific comparisons. Bacterial communities of vertebrates have also been found to be more similar among more closely related individuals than among distantly related individuals [36], [39], [65]. In the case of mammalian gut bacterial communities, however, diet had a greater effect on bacterial community structure than host phylogeny [36]. Together, these studies show that hosts can mediate their associated microbial communities, but that the influence of diet and other environmental conditions on the structure of bacterial communities can vary tremendously.

Our finding that insect diet has little effect on bacterial community structure may seem at odds with the substantial evidence that insects often form obligate associations with bacterial symbionts in response to nutrient-poor diets [66] and that insect diet has been found to alter insect-associated bacterial communities within specific insect species [67], [68], [69], [70]. However, distantly related insect symbionts of cicadas and sharpshooters (both sap-feeders) have converged on identical metabolic roles [60], demonstrating that the symbiotic functions bacteria provide to their insect hosts may not be phylogenetically constrained. If phylogenetically distinct bacteria can provide identical services to insect hosts, then diet would not be expected to correlate with bacterial community composition. Insects have been evolving for hundreds of millions of years, likely providing sufficient time for diverse insect symbionts to converge on similar symbiotic functions. While it is clear that diet can alter the bacterial community within an insect species, our cross-taxon study suggests that interactions between bacteria and the insect host have a greater effect than diet in governing community composition.

Our research, however, has two limitations that may restrict our ability to detect the effect of diet on bacterial community composition: 1) the broad diversity of insect samples, and 2) the use of whole insects. A study focused on a less diverse group of insects with varied diets may be better suited to resolve the effects of taxonomy and diet on bacterial communities. By using whole insects, we maximized our detection of insect-associated bacteria but also included endosymbionts (i.e. intracellular bacteria) in our analyses. Endosymbionts (e.g. *Buchnera*) often have profound effects on host nutrition, though in other cases (e.g. *Wolbachia*) they do not. Furthermore, because endosymbionts are predominantly transmitted vertically, they are under different selective pressures than environmentally acquired gut bacteria. Thus, while diet may affect both vertically transmitted and environmentally acquired bacteria, the signal from dietary effects on environmentally acquired bacteria would be obscured by vertically transmitted endosymbionts.

The cross-taxon analyses presented in this paper represent the first attempt to determine the effects of insect taxonomy and diet on insect-associated bacterial communities across a wide diversity of insects using relatively deep DNA sequence coverage and consistent methodology across all samples. Future studies could improve on this one by including greater insect diversity, comparing equivalent numbers of samples from each diet type, and using equal numbers of specimens from each species analyzed.

## Supporting Information

Table S1 **Classification of most abundant insect-associated bacterial phylotypes.**
(DOCX)
