# Supplementary material for: A Cross-Taxon Analysis of Insect-Associated Bacterial Diversity
Source: PLoS One. 2013 Apr 16;8(4):e61218. doi: 10.1371/journal.pone.0061218 (PMC3628706; doi:10.1371/journal.pone.0061218)
Supplement: Table S1 — Classification of most abundant insect-associated bacterial phylotypes. (DOCX) [file pone.0061218.s001.docx]

Supplementary Table 1. Most abundant bacterial phylotypes

|  |  | Average Relative Abundance | | |
| --- | --- | --- | --- | --- |
| Classification | # | All | present | Max |
| P;Alphaproteobacteria;Rickettsiales;Anaplasmataceae;Wolbachia | 17 | 14.8% | 34.0% | 90.9% |
| P;Gammaproteobacteria;Enterobacteriales;Enterobacteriaceae | 15 | 1.4% | 3.6% | 16.8% |
| P;Gammaproteobacteria;Enterobacteriales;Enterobacteriaceae;Enterobacter | 10 | 1.6% | 6.1% | 21.6% |
| Fi;Bacilli;Lactobacillales;Streptococcaceae;Streptococcus | 8 | 1.8% | 9.0% | 35.6% |
| P;Gammaproteobacteria;Enterobacteriales;Enterobacteriaceae | 8 | 0.7% | 3.2% | 9.8% |
| B;Bacteroidetes;Bacteroidales;Prevotellaceae;Prevotella | 7 | 1.7% | 9.6% | 27.8% |
| P;Gammaproteobacteria;Enterobacteriales;Enterobacteriaceae | 7 | 0.7% | 3.8% | 19.3% |
| P;Gammaproteobacteria;Enterobacteriales;Enterobacteriaceae | 7 | 0.5% | 2.9% | 9.2% |
| P;Gammaproteobacteria;Enterobacteriales;Enterobacteriaceae | 7 | 0.4% | 2.3% | 6.6% |
| A;Actinobacteridae;Actinomycineae;Actinomycetaceae;Actinomyces | 7 | 0.4% | 2.0% | 7.2% |
| P;Gammaproteobacteria;Pseudomonadales;Pseudomonadaceae;Pseudomonas | 6 | 0.6% | 3.8% | 11.3% |
| P;Gammaproteobacteria;Enterobacteriales;Enterobacteriaceae | 5 | 1.7% | 13.4% | 54.2% |
| P;Gammaproteobacteria | 5 | 1.3% | 10.1% | 24.6% |
| P;Alphaproteobacteria;Rhodospirillales;Acetobacteraceae;Gluconobacter | 5 | 1.2% | 9.3% | 25.4% |
| A;Actinobacteridae;Corynebacterineae;Mycobacteriaceae;Mycobacterium | 5 | 0.8% | 6.0% | 25.0% |
| U | 5 | 0.8% | 5.9% | 13.3% |
| B | 5 | 0.5% | 4.1% | 8.8% |
| Fi;Clostridia;Clostridiales;Veillonellaceae;Veillonella | 5 | 0.3% | 2.4% | 6.3% |
| P;Alphaproteobacteria;Sphingomonadales;Sphingomonadaceae;Sphingomonas | 5 | 0.3% | 2.3% | 6.1% |
| P | 5 | 0.3% | 2.3% | 7.2% |
| P;Alphaproteobacteria;Rickettsiales;Anaplasmataceae;Wolbachia | 4 | 2.3% | 22.7% | 44.8% |
| P;Alphaproteobacteria;Rickettsiales;Anaplasmataceae;Wolbachia | 4 | 0.8% | 7.5% | 15.7% |
| P;Gammaproteobacteria | 4 | 0.6% | 5.9% | 13.1% |
| P;Gammaproteobacteria;Enterobacteriales;Enterobacteriaceae; | 4 | 0.5% | 4.4% | 11.9% |
| P;Alphaproteobacteria | 4 | 0.4% | 3.9% | 9.8% |
| P;Alphaproteobacteria;Rhodospirillales;Acetobacteraceae;Asaia | 4 | 0.4% | 3.6% | 9.4% |
| P;Alphaproteobacteria;Rhodospirillales;Acetobacteraceae | 4 | 0.3% | 2.7% | 5.9% |
| P;Gammaproteobacteria;Pseudomonadales;Pseudomonadaceae;Pseudomonas | 4 | 0.3% | 2.6% | 6.0% |
| P | 4 | 0.3% | 2.5% | 5.5% |
| P;Betaproteobacteria;Burkholderiales | 4 | 0.2% | 2.0% | 5.9% |
| P | 3 | 2.7% | 35.6% | 74.4% |
| B | 3 | 2.5% | 32.2% | 85.3% |
| P;Gammaproteobacteria | 3 | 1.8% | 24.0% | 67.0% |
| P;Gammaproteobacteria;Enterobacteriales;Enterobacteriaceae;Buchnera | 3 | 1.2% | 15.1% | 41.4% |
| P;Alphaproteobacteria;Rickettsiales;Anaplasmataceae;Wolbachia | 3 | 1.1% | 13.8% | 33.6% |
| P;Gammaproteobacteria;Enterobacteriales;Enterobacteriaceae | 3 | 0.7% | 8.6% | 24.4% |
| P;Gammaproteobacteria;Pseudomonadales;Moraxellaceae;Acinetobacter | 3 | 0.4% | 5.8% | 12.0% |
| Fi;Bacilli;Lactobacillales | 3 | 0.4% | 5.2% | 11.0% |
| B;Flavobacteria;Flavobacteriales;Flavobacteriaceae;Empedobacter | 3 | 0.3% | 4.1% | 11.4% |
| Fi;Bacilli;Lactobacillales;Streptococcaceae;Streptococcus | 3 | 0.3% | 4.1% | 11.0% |
| B;Bacteroidetes;Bacteroidales | 3 | 0.3% | 3.3% | 7.9% |
| B;Flavobacteria;Flavobacteriales;Flavobacteriaceae;Chryseobacterium | 3 | 0.2% | 2.8% | 6.2% |
| P;Gammaproteobacteria;Enterobacteriales;Enterobacteriaceae | 2 | 1.6% | 31.0% | 49.8% |
| A;Actinobacteridae;Corynebacterineae;Corynebacteriaceae;Corynebacterium | 2 | 1.5% | 29.6% | 41.2% |
| P;Gammaproteobacteria;Enterobacteriales;Enterobacteriaceae;Providencia | 2 | 1.5% | 29.2% | 56.0% |
| P;Betaproteobacteria | 2 | 1.1% | 21.4% | 36.6% |
| B | 2 | 0.9% | 17.7% | 34.8% |
| Fi;Bacilli;Bacillales;Staphylococcaceae;Staphylococcus | 2 | 0.6% | 12.4% | 15.1% |
| P;Gammaproteobacteria;Pasteurellales;Pasteurellaceae | 2 | 0.5% | 10.0% | 18.4% |
| U | 2 | 0.4% | 8.6% | 16.4% |
| B;Sphingobacteria;Sphingobacteriales;Flexibacteraceae;Cardinium | 2 | 0.4% | 7.6% | 12.4% |
| P;Gammaproteobacteria;Enterobacteriales;Enterobacteriaceae | 2 | 0.3% | 5.8% | 11.4% |
| P;Alphaproteobacteria;Rhizobiales | 2 | 0.3% | 5.1% | 9.8% |
| P;Gammaproteobacteria;Enterobacteriales;Enterobacteriaceae | 2 | 0.2% | 3.9% | 6.4% |
| P;Alphaproteobacteria;Rickettsiales;Anaplasmataceae;Wolbachia | 2 | 0.2% | 3.8% | 6.9% |
| P;Gammaproteobacteria;Legionellales;Coxiellaceae;Rickettsiella | 2 | 0.2% | 3.3% | 5.1% |
| P;Alphaproteobacteria;Rhodospirillales;Acetobacteraceae | 1 | 2.5% | 95.6% | 95.6% |
| P;Gammaproteobacteria;Enterobacteriales;Enterobacteriaceae;Buchnera | 1 | 2.4% | 94.3% | 94.3% |
| P;Gammaproteobacteria;Enterobacteriales;Enterobacteriaceae | 1 | 1.4% | 54.9% | 54.9% |
| B | 1 | 1.4% | 53.6% | 53.6% |
| P;Alphaproteobacteria;Rickettsiales;Rickettsiaceae;Rickettsia | 1 | 1.3% | 50.0% | 50.0% |
| A;Actinobacteridae;Corynebacterineae | 1 | 0.8% | 32.8% | 32.8% |
| P | 1 | 0.8% | 31.8% | 31.8% |
| B;Bacteroidetes;Bacteroidales;Porphyromonadaceae;Dysgonomonas | 1 | 0.7% | 27.2% | 27.2% |
| B | 1 | 0.6% | 24.6% | 24.6% |
| P;Alphaproteobacteria;Rickettsiales;Anaplasmataceae;Wolbachia | 1 | 0.6% | 23.6% | 23.6% |
| P;Gammaproteobacteria;Pseudomonadales;Moraxellaceae;Psychrobacter | 1 | 0.6% | 22.3% | 22.3% |
| B;Bacteroidetes;Bacteroidales;Bacteroidaceae;Megamonas | 1 | 0.5% | 21.0% | 21.0% |
| P;Alphaproteobacteria;Rhizobiales;Phyllobacteriaceae | 1 | 0.5% | 19.1% | 19.1% |
| P;Alphaproteobacteria;Rickettsiales;Anaplasmataceae;Wolbachia | 1 | 0.5% | 18.6% | 18.6% |
| P;Alphaproteobacteria;Rickettsiales;Anaplasmataceae | 1 | 0.4% | 16.9% | 16.9% |
| U | 1 | 0.4% | 16.4% | 16.4% |
| A;Actinobacteridae;Micrococcineae;Brevibacteriaceae;Brevibacterium | 1 | 0.4% | 16.3% | 16.3% |
| A;Coriobacteridae;Coriobacterineae;Coriobacteriaceae;Collinsella | 1 | 0.4% | 16.2% | 16.2% |
| P;Alphaproteobacteria;Rickettsiales;Anaplasmataceae;Wolbachia | 1 | 0.4% | 14.0% | 14.0% |
| P;Alphaproteobacteria;Rickettsiales;Anaplasmataceae;Wolbachia | 1 | 0.4% | 13.7% | 13.7% |
| P;Betaproteobacteria | 1 | 0.3% | 13.2% | 13.2% |
| T;Mollicutes;Entomoplasmatales | 1 | 0.3% | 13.0% | 13.0% |
| B;Bacteroidetes;Bacteroidales;Porphyromonadaceae;Dysgonomonas | 1 | 0.3% | 12.3% | 12.3% |
| Fi;Clostridia;Clostridiales;Peptostreptococcaceae;Peptostreptococcaceae | 1 | 0.3% | 12.0% | 12.0% |
| B;Bacteroidetes;Bacteroidales;Porphyromonadaceae;Dysgonomonas | 1 | 0.3% | 11.4% | 11.4% |
| Fi;Bacilli;Lactobacillales;Lactobacillaceae;Lactobacillus | 1 | 0.3% | 11.1% | 11.1% |
| U | 1 | 0.3% | 10.9% | 10.9% |
| P;Gammaproteobacteria | 1 | 0.3% | 10.2% | 10.2% |
| U | 1 | 0.3% | 10.0% | 10.0% |
| B;Bacteroidetes;Bacteroidales;Porphyromonadaceae | 1 | 0.3% | 9.8% | 9.8% |
| B;Bacteroidetes;Bacteroidales;Bacteroidaceae;Bacteroides | 1 | 0.2% | 9.6% | 9.6% |
| U | 1 | 0.2% | 9.2% | 9.2% |
| B;Flavobacteria;Flavobacteriales;Flavobacteriaceae;Empedobacter | 1 | 0.2% | 8.4% | 8.4% |
| U | 1 | 0.2% | 8.4% | 8.4% |
| Fu;Fusobacteria;Fusobacteriales;Fusobacteriaceae;Sebaldella | 1 | 0.2% | 8.2% | 8.2% |
| Fi;Bacilli;Lactobacillales;Lactobacillaceae;Lactobacillus | 1 | 0.2% | 7.9% | 7.9% |
| P;Alphaproteobacteria;Rickettsiales;Anaplasmataceae;Wolbachia | 1 | 0.2% | 7.5% | 7.5% |
| Fu;Fusobacteria;Fusobacteriales;Fusobacteriaceae;Fusobacterium | 1 | 0.1% | 5.8% | 5.8% |
| U | 1 | 0.1% | 5.6% | 5.6% |
| P;Gammaproteobacteria | 1 | 0.1% | 5.5% | 5.5% |
| U | 1 | 0.1% | 5.4% | 5.4% |
| B | 1 | 0.1% | 5.3% | 5.3% |
| U | 1 | 0.1% | 5.2% | 5.2% |
| C;Chloroplast;Streptophyta | 1 | 0.1% | 5.2% | 5.2% |
| A;Coriobacteridae;Coriobacterineae;Coriobacteriaceae;Olsenella | 1 | 0.1% | 5.1% | 5.1% |
| B;Bacteroidetes;Bacteroidales;Bacteroidaceae;Bacteroides | 1 | 0.1% | 5.0% | 5.0% |
| P;Alphaproteobacteria | 1 | 0.1% | 5.0% | 5.0% |
| P;Epsilonproteobacteria;Campylobacterales;Campylobacteraceae;Arcobacter | 1 | 0.1% | 5.0% | 5.0% |

A: Actinobacteria, B: Bacteroidetes, C: Cyanobacteria Fi: Firmicutes, Fu: Fusobacteria, P: Proteobacteria, T: Tenericutes, U: Unclassified

#: Number of insect species in which phylotype is detected

All: Average relative abundance of phylotype across all insect species

Present: Average relative abundance of phylotype across insect species in which phylotype is present

Max: Maximum average relative abundance within an insect species
